# Using Linguistic Ethnography to Study Video Consultations: A Call to Action and Future Research Agenda

**DOI:** 10.1177/10497323221077297

**Published:** 2022-03-04

**Authors:** Lucas M. Seuren, Sara E. Shaw

**Affiliations:** 1 Nuffield Department of Primary Care Health Sciences, 6396 University of Oxford, UK

**Keywords:** technology, qualitative, doctor-patient, video-mediated interaction, linguistic ethnography, video consultations, research methods, linguistics

## Abstract

Video consultations are a rapidly growing service model, particularly in secondary care. Studies, mainly using trials and post-hoc surveys, have routinely documented that they can be a safe and effective means to deliver care at a distance. While video offers new opportunities to provide health services, it also constrains how patients and clinicians can interact, raising questions about feasibility, quality, and safety—questions that cannot be adequately addressed with prevailing methods and approaches. To support successful and appropriate implementation, use and spread of video consultations, we need to investigate how video changes the interaction. In this article, we use two worked examples to demonstrate how Linguistic Ethnography, a methodological approach combining ethnographic with linguistic analysis, enables a detailed understanding of how communication in video consultations works, providing an evidence base to support patients and clinicians with using this service model.

## Introduction

Video consulting (using Teams, AccuRx, and other media) is a rapidly growing service model in healthcare. The COVID-19 pandemic has brought requirements for social distancing to reduce the spread of a highly infectious disease that have forced clinicians to limit in-person consultations whenever possible. This has led to video consulting, previously a niche activity, having a bigger role in health care delivery (Greenhalgh, Wherton, Shaw, & Morrison, 2020), especially in secondary care (e.g., Gilbert, Billany, et al., 2020; Paleri et al., 2020).

Integrating video consultations in routine clinical practice is not easily achieved. In addition to organizational and infrastructure challenges (Greenhalgh et al., 2020), video consultations require different communication skills from clinicians and patients (Roberts & Osborn-Jenkins, 2020), and the medium itself means that the distribution of labor is different (Gilbert et al., 2020b), for example, patients or carers might now perform a remote physical examination (Seuren et al., 2020).

Video is similar to in-person in that most (though not all) patients and professionals can use it, however the technology changes the interactional dynamics. Video technology, like any communication medium, has *affordances* (Hutchby, 2001): it provides potential opportunities and challenges for interaction. For example, while video makes it possible to actually see patients remotely, which can help maintain the therapeutic relationship (Ignatowicz et al., 2019), that view is often restricted to their head and shoulders, which makes it hard to see and use gestures (Connolly et al., 2020).

The literature on video consultations is growing rapidly (Connolly et al., 2020; Ignatowicz et al., 2019; O'Cathail, Sivanandan, Diver, Patel, & Christian, 2020), but we have a limited understanding of what actually goes on in a video consultation, what makes it different from an in-person appointment, and what skills clinicians and patients need. Although Pappas and Seale (2009; 2010) began to investigate the interaction in video consultations over 10 years ago, it is only recently that more researchers have begun to look at these issues (e.g., Due & Lange, 2020; Ilomaki et al., 2021; Stommel & Goor, 2019; Stommel & Stommel, 2021). To support the use of video consultations in health services we need to know when video technology might appropriately be used. This means investigating not only what the affordances of the technology are, but also *how* they affect the consultation, that is, how patients and professionals communicate over video and how participants do and do not successfully achieve their goals for a particular consultation (Shaw et al., 2020).

This article aims to demonstrate why combining ethnographic methods with fine-grained, (multimodal) linguistic analyses of recorded video consultations is critical for understanding video consulting, for informing appropriate communication strategies for participants, and for supporting appropriate spread of video consulting. We argue that Linguistic Ethnography (LE) is one potential methodological framework for achieving this goal: it facilitates focus on the interplay between social life and the inner workings of communication. This enables researchers to simultaneously gain a fine-grained appreciation of how participants accomplish technology-mediated interaction and how this shapes and is shaped by the wider context in which video consulting is rapidly evolving.

We first discuss the current state of research on video consultations, including the use of deductive approaches to study interaction. We argue that such studies only provide a partial picture of video consultations. We then set out how linguistic ethnography can enable an in-depth understanding of video consulting and providing two worked examples from our recent research. We close by discussing the practical challenges of using LE to study video consulting and setting out an agenda for future research.

### Overview and Limitations of Current Research on Video Consultations

There is now a vast body of work investigating the feasibility, acceptability and practicalities of video consultations in a range of clinical settings. Findings have largely been positive, reporting that video consultations are (on the whole) safe, effective and acceptable to both patients and clinicians, serving as an adequate complement or even alternative to in-person consultations or other forms of telemedicine (e.g., telephone consultations) (Connolly et al., 2020; Ignatowicz et al., 2019; O'Cathail et al., 2020).

These studies have provided valuable insights into the work involved in video consultations. However, the majority typically focus on testing the technology, using quantitative methods such as clinical trials and surveys. In this section, we review the dominant methods used to date and what the data they produce tell us about video consulting.

### Studies of Feasibility and Acceptability of Video Consulting

To date, the majority of research on video consultations has explored patient and clinician perceptions of feasibility and acceptability. Studies generally involve small-scale trials that investigate whether and under what conditions video consultations are acceptable in a specific clinical setting. After conducting a video consultation, patients and clinicians complete a survey with closed questions (e.g., likert-type scales) and open-ended questions. In this way, they document their experiences and what they see as the benefits and challenges of video. In some studies, researchers use semi-structured interviews to develop a richer understanding of participants’ perspectives (e.g., Donaghy et al., 2019; Gilbert et al., 2021; Triantafillou et al., 2020; Wade et al., 2014).

These studies have been valuable in demonstrating widespread feasibility and acceptability of video consultations across specialties—for example, in diabetes (Fatehi et al., 2015) or orthopedic rehabilitation (Gilbert et al., 2021)—depending on clinician and patient preferences and circumstances and on the suitability of each consultation for use of video (e.g., where no physical examination required, Armfield et al., 2015). However, they are limited in scope. Each study tends to focus on a single clinical setting, trial design tends to focus on “testing” the technology rather than development, implementation and routinization of the service model, and semi-structured interviews and surveys provide only a partial picture of respondents’ views.

Some studies have indicated the potential of video consulting to change the interaction between patients and clinicians and to enable a high quality consultation (Hammersley et al., 2019). To study this, we need methods that can access and appreciate the interaction. Participant responses in interviews and surveys are recollections of what went on during a video consultation (Silverman, 2004, 2017; Whitaker & Atkinson, 2019). This process of collecting data fails to access the largely taken-for granted norms and routines that shape interaction in a video consultation. Take the example of non-verbal communication cues (e.g., gaze direction). Interview respondents have raised a concern that such cues are missed in video consultations (Miller, 2003), but it is impossible to tell from interview data which cues are missed or how this shapes the interaction and outcome of the consultation. Moreover, studies of non-verbal behavior reveal that people’s reported experiences do not always correspond to “the real world” (e.g., clinicians often believe that in video consultations they look at patients more than they actually do, Faucett et al., 2017).

Human social actions are constructed through a complex combination of semiotic resources (Goodwin, 2000). To gain a deeper understanding of what goes on during a video consultation—for example, what actions participants perform, how they perform them, what kind of problems they experience—and how the interaction is shaped by the technology and wider infrastructure, methods are needed that allow real-time, naturalistic observation of video consultations.

### Research on Video Consulting using Deductive Interaction Analysis

Research on interaction and communication in video consulting does exist. Published studies have typically used deductive coding systems (e.g., Roter Interactional Analysis System, RIAS, Roter & Larson, 2002; Davis Observation Code, Callahan & Bertakis, 1991), enabling researchers to classify each utterance in an interaction and quantify the types of talk.

Take our earlier work in which we used RIAS to compare video with in-person consultations in three clinical settings (diabetes, antenatal diabetes and hepatobiliary cancer) (Greenhalgh et al., 2018). We used RIAS to classify each utterance from 37 video and 28 in-person consultations as accomplishing a specific action (socioemotional, task-focused, process oriented or technology-related) and then used statistical analysis to investigate how video and in-person consultations differed in consultation length, the types of non-technology focused talk, and participant dominance. We found that video was similar to in-person, with video consultations slightly shorter and antenatal diabetes consultations slightly more clinician-dominated via video than in-person (Greenhalgh et al., 2018). Findings proved helpful in drawing attention to potential differences in modalities and in drawing up guidance to support this new service model. However, use of such deductive systems has limitations. We focus on two of these (see e.g., Sandvik et al., 2002 for a detailed account).

First, deductive coding systems only allow researchers to see data within the constraints of that coding system. New or unexpected actions or structures of action have to be coded according to the existing system, limiting researchers to what they already (presume to) know about the working of social interaction. This is a salient issue for video consultations. Deductive coding systems were designed for analyzing in-person consultations (Agha et al., 2009; Hammersley et al., 2019), but technology-mediated interaction is not simply a different form of in-person interaction: participants adapt their communication strategies and develop new ones to deal with the technology (Arminen et al., 2016). Use of coding systems designed for in-person interaction therefore potentially restricts researchers, and likely focuses on the restrictions of video. Attempts have been made to address this (e.g., modifications of RIAS encourage consideration of the features of telemedicine such as talk about privacy and security, Miller & Nelson, 2005). However, these have not been informed by detailed analyses of telemedicine, but rely on reported experiences from surveys, interviews and panels, and assumptions about what matters in telemedicine.

Second, deductive coding systems do not adequately account for non-verbal interaction (Miller & Nelson, 2005) or the wider context of video consultations. This is important as, to understand how video consultations work, researchers need to account for gestures, gaze and body position, and the use of objects like electronic patient records or the technology itself. These are difficult to code and are therefore rarely included.

In sum, current evidence on video consulting draws largely on (small-scale) feasibility trials and indirect methods (surveys, interviews) to understand the experiences and expectations around video consulting; and on deductive coding systems to analyze interaction. These methods are limited. They provide little (if any) opportunity for detailed understanding of the interactions, norms and routines that make up a video consultation.

### Combining Linguistics and Ethnography to Study Video Consulting

Linguistic Ethnography is a methodological approach that uses mainly qualitative approaches to enable intensive analysis of language and communication in ways that shed light on small, but consequential, aspects of social practice (Rampton et al., 2004). LE has strong ties with the North American tradition of Linguistic Anthropology, which is similarly focused on the relation between language, society and culture and hence shares many of the same antecedents (Goffman, 1959; Gumperz & Hymes, 1986). While continuities with linguistic anthropology remain, LE has emerged as a largely European phenomenon as scholars combining linguistics and ethnography—and increasingly concerned with interdisciplinarity—sought to find a home (Copland & Creese, 2015).

This set of European circumstances prompted the foundation of Linguistic Ethnography Forum in 2001, through which “a number of key scholars and lines of enquiry were pushed together by circumstance, open to the recognition of new affinities, and sufficiently familiar with one another to treat differences with equanimity” (Rampton, 2007, p. 585). What has emerged is not a singular method or theoretical framework, but “cluster of research” (Maybin & Tusting, 2011) that shares epistemological roots, is wide-ranging in its empirical scope and provides an intellectual space that embraces a range of interpretive and discursive approaches (Copland & Creese, 2015; Maybin & Tusting, 2011; Rampton et al., 2004; Tusting, 2019). A range of methods and approaches come under the umbrella of LE, including Conversation Analysis (Maynard & Heritage, 2005), Ethnography of Communication (Gumperz & Hymes, 1986), Discourse Analysis (Sarangi, 2010) and Multimodal analysis (Bezemer et al., 2017) (see Tusting, 2019 for a thorough overview). Whatever the approach adopted, the broad aim is to develop an emic understanding of how people communicate in particular contexts and settings.

LE researchers typically unite ethnographic methods (e.g., observation, interviews) with close inspection of interactional data. This enables understanding of both how interactions work and the socio-cultural and organizational contexts in which they take place (Tusting, 2019). In relation to video consulting, LE analysis involves an iterative process of “zooming in” on the individual interactions in video-mediated consultations and “zooming out” to the interpersonal, clinical and policy context (Shaw et al., 2020). From an LE perspective, the aim is to develop a comprehensive understanding of how video consultations are conducted, and how they shape clinical routines and practices, as well as wider organizational, technical, policy and professional infrastructure. There are inevitable differences (e.g., a concern in Conversation Analysis with the structural organization of social interaction—typically taken to be independent of the broader context (Schegloff, 1997)—sits uncomfortably alongside the ethnographic concern with the social context in which that same interaction takes place). However, this combination of ethnography and linguistics offers an invaluable tool to studying video consultations. Below we set out four reasons why this is the case. In the subsequent section, we present two worked examples from our research.

The first way in which LE can contribute is by demonstrating how the context in which each video consultation takes place affects the way patients and clinicians communicate in a video consultation. This is characteristic of LE, with ethnography used to “open up” the analysis and linguistic analysis used to “tie it down” (Rampton et al., 2004). Ethnographic methods (e.g., observation, interviews) enable understanding of the broader context (e.g., procurement of different video technology platforms, booking systems for patient appointments, professional indemnity), which shapes how consultations work. In LE they therefore provide an invaluable resource for understanding “the tasks, goals, and practical problems at hand in particular health care interactions” (Leydon & Barnes, 2020, p. 144). It is then the combination with linguistics in LE that aids appreciation of how participants attend (or not) to these contextual features. It is this combination that allows LE researchers to show how the context matters for, and is made to matter by, participants during actual interactions, that is, how it is “procedurally consequential” (Schegloff, 1991).

Second, linguistic ethnography encourages investigation of the mechanics of social interaction (i.e., norms, conventions, and practices), including video-mediated interaction (Arminen et al., 2016; Mlynář et al., 2018). As such it offers an empirically validated theoretical lens, or “toolkit” (Schegloff, 1988), for analyzing and understanding video consultations.

Third, linguistic ethnography applies inductive and data-driven approaches focused on everyday use and experience that can reveal aspects of video consultations and their context that researchers may not expect to find or think worthy of study. People structure social interaction in ways that are generally “seen but unnoticed” (Garfinkel, 1964), meaning that researchers do not always know a priori which questions will be relevant to ask. It is only by problematizing what may at first seem trivial—“making the familiar strange”—that we can understand the implications of these taken-for-granted routines for the smooth flow of our daily interactions. In the case of video consultations, this unmotivated perspective is likely to be important: video is still a relatively new modality for communication and video consulting a fairly new service model. Both service users and researchers may have a limited idea about what would constitute normal behavior in a video consultation.

Fourth, by grounding analysis in an understanding of how social interaction actually works, instead of common-sense assumptions about how interaction supposedly works, linguistic ethnography can generate beneficial societal impact (Copland & Creese, 2015). In the case of video consulting, this might involve development of guidance and support for clinicians and patients (Wherton et al., 2020).

### Applying Linguistic Ethnography in Studies of Video Consulting

In this section, we present two worked examples from our research on video consulting to illustrate the potential of linguistic ethnography. We draw on secondary analysis of video consultation data from two studies (see Greenhalgh et al. (2018); Shaw et al. (2018); Shaw et al. (2020) for further details), combining data from 26 patient interviews and 35 staff interviews, with documents, field notes and demographic data, along with 37 video-recorded video consultations and 28 audio-recorded in-person consultations in four clinical settings (diabetes, antenatal diabetes, cancer, and heart failure).

Our methodological approach combined Ethnography of Communication and Conversation Analysis. The former is concerned with how context shapes communication, focuses on the cultural and organizational knowledge that forms the basis for specific communicative events (e.g., consultations) and examines *Communicative Competence* (i.e., what a speaker needs to know to communicate appropriately within a particular speech community) (Saville-Troike, 2003). The later offers an excellent complement focusing on how patients and clinicians collaboratively accomplish the consultation (Halkowski & Gill, 2010; Leydon & Barnes, 2020). It takes the sequential and indexical organization of talk-in-interaction (its turn-by-turn production) as its starting point; interrogating how each turn is fitted to the prior talk, what kind of response it projects, and the verbal and non-verbal behavior (or “practices”) by which this is accomplished (Sidnell & Stivers, 2012). The driving question is how participants publicly and accountably make their verbal and non-verbal actions recognizable and understandable for each other and how they make sense of each other’s actions.

Ethnography of Communication and Conversation Analysis, have similar ontological and epistemological roots: they were developed in parallel in the 1960s and 1970s as constructivist perspectives to study language-in-use, with the aim of understanding how language and the context of interaction matter for participants. In spite of the friction that exists in their approaches to context (Schegloff, 1997), combining them is valuable on two levels. First, we need ethnography to make sense of interactional data from unfamiliar settings. Maynard (2006) points out that CA and Ethnography have what he calls “limited affinity.” As researchers, we generally lack the training and expertise of clinicians or the lived experience of patients. Ethnographic data provides an invaluable resource for helping researchers to access and understand the social, material, technological, environmental and operational issues at hand in video consultations and to analyze the interactions.^1^

Second, to develop useful guidance or policy it is critical to understand the broader social and cultural context: for example, procurement of different video technology platforms, booking systems for patient appointments, professional indemnity. Ethnography can help us to assess the challenges and benefits of video consultations in regards to these wider social issues, with CA aiding appreciation of how participants attend (or not) to these contextual features.

Studies from which data were drawn received ethical approval from the National Research Ethics Committee London-City Road and Hampstead in December 2014 (14/LO/1883) and by the South Central-Berkshire Research Ethics Committee in September 2015 (15/SC/0553). All participating staff and patients provided informed consent for audio and video recording and for data to be used for research.

### Worked Example #1—Technological Trouble and Implementing Video Consultations

Video consultations may seem straightforward, with clinicians simply using video technology to call their patients in much the same way as they use the telephone. But as we documented in previous ethnographic research (Greenhalgh et al., 2017), video consulting is part of a complex socio-technical system in which the information infrastructure—meaning the software, hardware, organization, rules, regulations etc.—needs to adequately support the development, set up and on-going maintenance of remote healthcare delivery (Sittig & Singh, 2010). For instance, communication software such as Microsoft Teams or AccuRx needs to be integrated into existing clinical routines as well as technological infrastructure; staff and patients need to be supported to change the ways in which they interact and work; and incompatibilities across systems need identifying and addressing (Greenhalgh et al., 2018).

The use of video consultations—and interaction in video consultations—is shaped by this evolving, and highly challenging, context. For example, in our research staff at two clinical sites (part of the same NHS Trust), used Skype to conduct video consultations. While seemingly straightforward (e.g., agree service design, install software, set appointment, call patient and so on), staff faced repeated material, technological and operational issues that were not easily overcome, and were frequently compounded by wider organizational constraints (e.g., outdated IT infrastructure, status as a “pilot service” meaning that staff were unable to directly install or manage updates themselves, Greenhalgh et al., 2019). As field notes from a video consultation in oncology show, this organizational and technological infrastructure had consequences for how video consultations ran:

The audio was not working properly again. SB and SR called me in to help out, but there was little I could do to rectify it. The video quality was fine but there was no audio – neither side could hear. I felt powerless to help, as their audio (microphone and speaker) was on and full volume. The call was shut down. They decide to run Skype (for video) and SR’s mobile phone on loudspeaker (for audio) simultaneously

As the above extract suggests, a frequent infrastructural issue in video consulting is insufficient bandwidth or inadequate Wi-Fi coverage, resulting in *latency* or *lag* (i.e., delays between one person talking and the recipient hearing them). In our research this was identifiable, not only in our ethnographic data, but also our interactional data, with latency making it interactionally challenging for participants to maintain the conversational flow or turn-taking, that is, how they determine whose turn it is to talk (Sacks et al., 1974).

To study this more closely we have begun to develop a systematic analysis of how the conversational flow in video consultations is affected by latency (Seuren et al., 2021). We conducted secondary analysis of 25 video consultations for which we had video recordings at both the patient’s and the clinician’s end of the call. We transcribed these and then compared the two sides of each call, focusing on (a) problematic silences that occurred after a speaker completed a turn, and (b) moments where patient and clinician talked at the same time. In line with the emic perspective of Linguistic Ethnography, we considered a silence “problematic” if either participant used dedicated communication strategies to solve it. Data from interviews and observations allowed us to make sense of the ways in which staff and patients understand and negotiate silences and overlaps. We demonstrated that participants routinely struggle, because they cannot adequately make sense of silence—often understood as indicating a problem with the organization of the talk (e.g., the speaker was not heard or understood), and requiring “repair” strategies (Schegloff et al., 1977). As is usual in LE, this allowed us to connect with existing theory on the organization of silence and overlapping talk, which has been foundational for conversation analysis (Hoey, 2020; Sacks et al., 1974; Schegloff, 2000).

**Figure 1.**
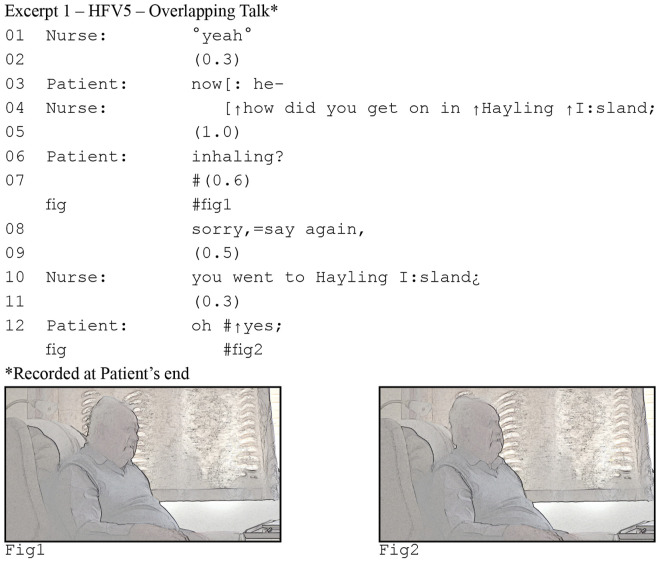
Patient's perspective of delayed response .

**Figure 2.**
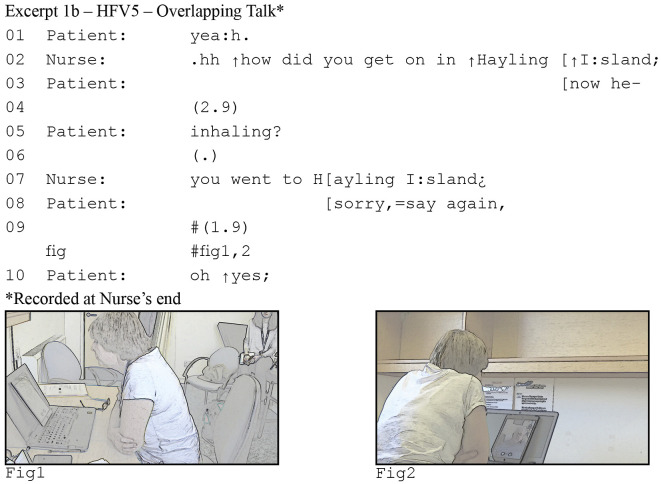
Nurse's perspective of delayed response

Focusing in on the interactional data, consider the following two excerpts (Figure 1 and 2) from a consultation between a heart failure patient and his specialist nurse. The nurse asks a question in line 4, but the patient mishears “in Hayling” as “inhaling.” He initiates repair in line 6 by repeating what he thinks he heard, a conventional practice for dealing with hearing problems (Robinson, 2013).

Our focus is on lines 6–8. The patient’s repair initiation makes relevant a response; it is what we would call a “sequence-initiating action” or “first pair part” (Sacks et al., 1974). By repeating part of her question, the patient reveals what he heard, and thereby requests the nurse to repeat the part he did not hear. In English conversations, responses generally come quickly and silences, if any, are brief: on average no more than 200ms (Stivers et al., 2009). This means that when we find a longer silence, it is not just silence, but it can be understood by participants as the noticeable and meaningful absence of a response. Here, the patient breaks the silence by explicitly asking her to repeat her question, thus taking that 600ms silence as an indication that the nurse is not going to answer.

At first glance excerpt (1a) is unremarkable. Response pursuits such as the one produced by the patient occur routinely in in-person conversations. However, if we consider the nurse’s end of the call, a different perspective emerges. In excerpt (1b), we see the same stretch of talk, but notice now that there is less than 200ms silence between the patient’s repeat “inhaling” in line 5 and the nurse’s response in line 7. In other words, the nurse responds “on time” (Sacks et al., 1974). We also see that for the nurse, the patient’s response pursuit arrives while she is speaking. The reason the patient in (1a) perceives such an extended silence, is the latency in the consultation. However, the patient does not notice or realize this. He thus pursues a response, when the nurse has already begun to provide one.

The combination of interactional and ethnographic data was key in enabling us, not only to see how problems with turn-taking arise and are resolved, but also how they are explained and accounted for through the mechanics of social interaction. Interviews following the consultation, for instance, showed that participants do recognize turn-taking problems (even if they do not articulate it as such) and that interactional problems matter for them. Take the following example from a community heart failure nurse explaining her concern that latency in the video connection means she is not sure if the patient is picking up what she’s saying (and vice versa).

It’s getting used to it, isn’t it. Because you’re saying something, and he’s saying something, and then you think he’s finished, and he hasn’t finished. And of course when you’re together, you talk like that, don’t you. Because you wanna move it either on a bit or you want to clarify a bit that they just said and stuff. But whereas of course with it- when you’re not together, it’s harder, isn’t it. Cause you’re not sure who’s picking up what really.

It is through such interview and observation data that we began to grasp how the problems of turn-taking are noticed by participants.

We know from our ethnographic data and other studies (e.g., Ilomaki et al., 2021) that participants are concerned with the conversational flow in video consultations. Most participants, both patients and clinicians, used mobile devices (smartphones, tablets, or laptops—sometimes in combination). While the mobility of these devices facilitates video consultations, for example, when doing a physical examination (see worked example #2), their use meant that patients and nurses relied on their wireless networks and connections, which are inherently slower and less stable than wired network connections. By combining contextual appreciation (through ethnography) with detailed analysis of the interaction (through Conversation Analysis), our analysis shows how latency impedes the ability of patients and clinicians to maintain the conversational flow: they may be silent at points where they should be talking, and may routinely interrupt each other.

This evidence enabled us to develop new guidance. One proposed solution for latency was for clinicians to allow for *longer* silences when the patient stopped talking (Faucett et al., 2017). Our analysis of the interaction, however, indicates that when silence is due to latency, recommending that clinicians wait longer after the patient finishes their turn is likely detrimental to the quality of the consultation. As we see in excerpt (1a), when silences become “too long,” they will often be understood to indicate an interactional problem, such as a mishearing or misunderstanding (Kendrick & Torreira, 2015), or as foreshadowing a non-straightforward answer (Robinson, 2020). Part of the solution for the compromised conversational flow due to latency is not for participants to wait longer when their co-participants finish talking, but to wait longer when they themselves finish, so they can be sure that their co-participant has not already started.

We did not start out our investigation with latency in mind. It was through review of both ethnographic and interactional data that we began to understand the potential relevance of micro delays. We used these initial ideas to develop a more systematic approach, zooming in on the specific problems and communication strategies that characterize conversational flow in video consultations, and zooming out to the wider context which these consultations take place to appreciate staff and patient perspectives and the organizational and technological set up of video-mediated heart failure consultations.

### Worked Example #2—Using Technology to Conduct Physical Examinations via Video

In video consultations, clinicians cannot lay hands on the patient. Nonetheless, physical examinations are sometimes conducted by video, for instance to review a skin rash (Marchell et al., 2017), monitor blood pressure (Greenhalgh, Koh, & Car, 2020) or conduct physical therapy (Gilbert et al., 2020b). This has raised questions about what kinds of physical examination are possible by video, what kind of support participants need (e.g., a carer helping the patient), and what (verbal and non-verbal) communication skills participants at both ends need to successfully conduct one (O'Cathail et al., 2020).

We have recently begun to investigate how physical examinations are carried out by video (Seuren et al., 2020), focusing on video-recordings of seven heart failure consultations along with field notes and interviews with the patients, carers and clinicians involved. These consultations included an assessment for ankle edema in which the patient or carer needed to press their thumb into the patient’s lower leg and ankle to see if that left an indentation (indicating fluid retention and reduced blood flow). We investigated how each examination was conducted, focusing on (a) how clinicians give instructions or guidance, (b) how patients or carers responded to these instructions, and (c) how and where patients or carers raised problems with the examination.

Findings showed that while these examinations can be accomplished remotely, patients and carers struggle to conduct the examination while at the same time making it visible to the clinician using their device (smartphone, tablet or laptop). Clinicians therefore found it hard to make visual assessments of the patient’s body, and had to rely on the patient’s or carer’s assessment instead. This was neatly articulated by one community heart failure nurse, describing how she felt the remote physical examination went:

Difficult. Because from my end, it looked like she didn’t have any fluid. But she said, “oh well they’re not like they were before; they’re a lot better.” Rather than saying, “oh well the fluid’s gone completely.” I think she thought she still had some swelling in her feet. But I couldn’t judge that at all.

**Figure 3.**
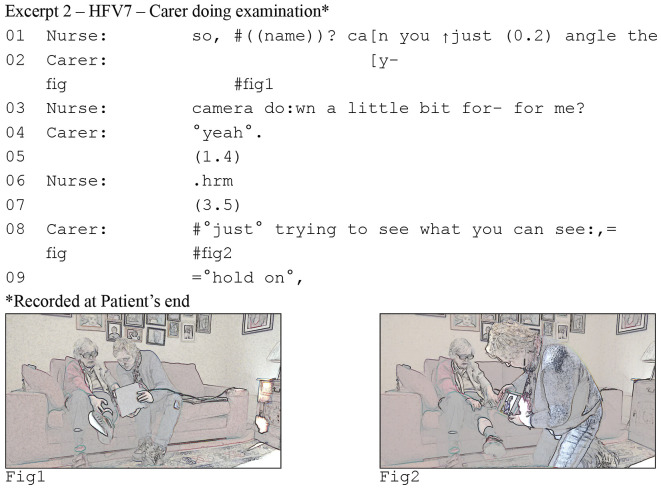
Carer struggling with performing a physical assessment

As the above extract illustrates, interview data helped us to understand where clinicians saw as a discrepancy between what the patient told them and what they could see. By zooming in on the interaction, we then got an in-depth understanding of how these problems emerge. Consider excerpt (2) (Figure 3). The patient is with a carer and they are using a tablet to conduct the consultation. The nurse has asked the patient whether she has fluid in her legs, that is, whether she has edema. In response, the patient has rolled up the leg of her trousers and her carer is aiming the tablet at the patient’s leg (as can be seen in #screengrab1). However, the nurse cannot adequately see, and asks the carer to change the angle (lines 1–3). The carer then changes her position and that of the tablet (see #screengrab2), making clear that she is “trying to see what [the nurse] can see” (line 8).

This excerpt reveals a problem with video examinations where participants attempt to overcome the contextual restriction that clinicians cannot lay hands on the patients. The patient and carer need to perform the examination instead, and they need to show the clinician what they are doing—only the clinician has the training and expertise to make a clinical assessment. However, they cannot adequately monitor what the clinician can see and instead have to rely on instructions and feedback from the clinician (e.g., see lines 1–3). In a video call, a small screen-in-screen shows people what their co-participant can see. Once the carer aims the camera away from herself, she loses track of that screen. She has the tablet at a slight angle, forming a sort of triangle with herself and the patient’s leg. In this way, she can still see what the camera is recording, but she aims the camera at the patient’s hip in #screengrab1 and above the patient’s leg in #screengrab2.

The carer continues to struggle to show the patient’s leg to the nurse. The carer eventually manages to show the patient’s leg after about a minute of maneuvering, but she does not manage to conduct the examination while simultaneously showing it to the nurse. As a result, the nurse has to rely on the carer’s “lay” assessment that the patient does not have edema.

One could conclude after this consultation that these physical examinations for edema are not feasible by video, even with the support of a carer and the flexibility of a tablet. And while the carer reported in the interview after the consultation that she was “fannying about” to get a picture of the patient’s leg, she did offer a solution:

But it’s partly, because I’m holding it, and I can’t see what I’m looking at. Whereas if you put it down, and you see, oh well yeah that’s a way off, now I can, you know, so, you can see immediately.

By conducting a data-driven analysis, we see how other patients and carers use different strategies to overcome this problem, and some of those are in fact successful.

**Figure 4.**
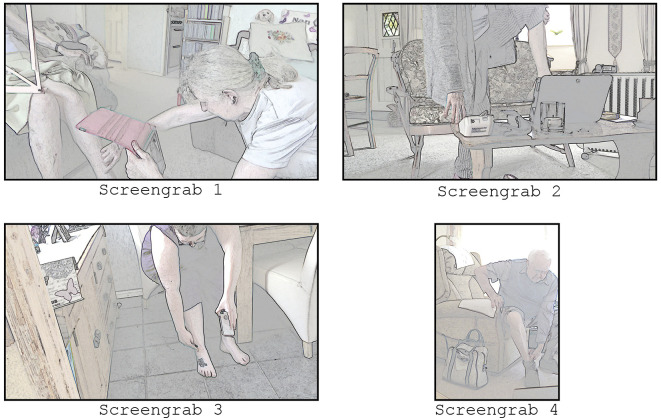
Screengrabs from video examinations during four different heart failure consultations.

Consider the screengrabs in Figure 4, from four different heart failure consultations. The participants use different types of technology (smartphone, tablet, laptop), which affects how they conduct the examination. Smartphones and tablets can be moved around, whereas a laptop needs to be in a fixed position requiring more work, and thus mobility, from the patient. The carer in screengrab 1 uses the camera on the back of her tablet to show what she is doing, and successfully completes the examination of the patient’s legs. The patient in screengrab 2 can show her leg by holding it up to the camera, but this comes with its own risk: she stands on one leg, which could have caused her to fall. The patient in screengrab 3 uses a smartphone, which has the advantage that she can move it around without assistance, but she has to rely on the clinician’s feedback to determine how to hold her phone. Finally, the patient in screengrab 4 placed his laptop on the floor, allowing him to examine himself safely, while also being able to monitor what the camera is recording. Technology is only one factor shaping the remote examination. Guided by LE, zooming out to the clinical and social context allows a more comprehensive analysis of these different technologies. Combining detailed analysis of interactional practices with an appreciation for the clinical context allowed us to develop a more comprehensive analysis of how video consultations are conducted and what is potentially feasible.

In sum, linguistic ethnography proved crucial in allowing us to (i) show how the video-mediated context changes how physical examinations can be done, and that despite concerns raised in interview and survey studies, in some cases and for some patients they can be done; (ii) draw on foundational work on multimodality (e.g., Mondada, 2016) to make visible the verbal and non-verbal communication practices that participants use to accomplish these physical examinations; and (iii) use analysis of both ethnographic and interactional data to show how participants struggle to conduct the examination and make it visible at the same time, and how participants use technology to overcome this problem. Findings provided an empirical evidence base for patient and clinician guidance on the use of physical examinations via video (Wherton et al., 2020).

## Discussion

With video consulting becoming an important service model in response to the COVID-19 pandemic it is crucial that clinicians and patients have appropriate evidence and support to help them use it. This requires a comprehensive understanding of video consulting. Traditional research approaches using trials, surveys and interviews have demonstrated feasibility and acceptability. A wider range of research designs and methods is urgently needed in order to access the social, organizational and clinical context in which video consultations take place, and the interactional routines through which clinicians and patients accomplish them. This requires studies that examine the real-life complexities of implementing and using video consultation services. In this article we have (i) set out a strong argument for the value of Linguistic Ethnography as an overarching methodological approach to studying video consulting that focuses on the context and structural organization of social interaction, both in and outside healthcare; (ii) used that framework to provide illustrative examples combining ethnographic methods with a fine-grained analysis of communication in video consultations; (iii) demonstrated the criticality of this approach for generating understanding about how the use of video technology shapes the clinical encounter and how and when it is feasible and appropriate; and (iv) shown how detailed observation and rigorous analysis of video consultations, guided by linguistic ethnography, can inform practical guidance to support video consulting.

There is a range of other methodological approaches to thinking about and analyzing interaction, each with strengths and weaknesses. This includes, for instance, Linguistic Anthropology (LA), situated in North-American anthropology and focused on the cultural settings and activities in which people use language (Duranti, 2009). LA combines ethnographic and interactional analytic techniques but tends to take a more macro perspective to examine the structure, use and evolution of language (Enfield et al., 2014). Its investigation of language and culture using qualitative methods makes it particularly suitable for addressing questions about how video consulting is embedded in healthcare systems and how this shapes and is shaped by the interaction. Video(-Reflexive) Ethnography (VE and VRE) uses video-recording and editing processes, working actively with clinicians and/or service users to shed light on social and organizational processes (Collier & Wyer, 2016; Iedema & Bezemer, 2021). Analysis does not involve detailed linguistic analysis, but instead offers a form of visual scholarship that guides researchers’ attention to the spatial and embodied dimensions of interaction and collaboration in healthcare. There is no one size fits all methodological approach here: each offers strengths and challenges. We encourage researchers, and those funding and supporting research, to acknowledge the importance of examining social context and interaction and to then take up the approach most suited to the question/s at hand.

Studies using linguistic ethnography and similar approaches are rarely straightforward. Conducting a qualitative analysis using observations, interviews and video-recordings of consultations is labor intense, provides a range of practical and ethical challenges and requires specific methodological and analytical expertise from researchers. As with any observational study of healthcare interaction, the work is even more challenging during a pandemic (e.g., involving use of personal protective equipment when visiting sites, or social distancing with participants). We often need to rely on remote recording techniques (e.g., using screen capture software combined with secure services to transfer data from local sites). Such methods have practical and ethical implications, not least given the additional burden on patients and clinicians. Even in post-pandemic times, visiting sites may not always be feasible. Close inspection of interaction requires researchers to make video recordings of the communication setting. As discussed by Parry et al. (2016), video-based research raises concerns around acceptability—are patients willing to be recorded for research and training—as well as risk to quality of care, confidentiality, and coercion of vulnerable people. Any study design needs to consider these risks and provide measures, documented in a research protocol, to mitigate against them (e.g., detailed and careful procedures around informed consent). In such cases, while research informed by linguistic ethnography may remain the ambition, researchers may need to think creatively about how they can adapt research designs and methods. Whatever the case, fine-grained studies that involve direct observation and appreciation for their context of use, are necessary to fully appreciate the complexity of video consultations (Halkowski & Gill, 2010).

There is already a small but growing body of research using interactional and ethnographic approaches to understand video mediated communication within and outside of healthcare (Dalley et al., 2020; Mlynář et al., 2018). Given the prominence of video consulting in health care during (and likely beyond) the COVID-19 pandemic, future research will need to take communication in video consultations as a more central analytical focus. We propose four key areas where such research should focus.

First, research on how interaction in video consultation works must consider how it is shaped by the affordances of the technology—its opportunities for new ways of interacting as well as its restrictions for achieving the goals of a video consultation (Arminen et al., 2016; Hutchby, 2001). Participants have access to various kinds of technology (e.g., smartphones, tablets, laptops, desktop PCs), that vary in terms of mobility, screen size, camera quality and so on. Each option will have its own benefits and challenges. For example, with some communication solutions, clinicians can switch from a telephone consultation to a video consultation in real-time if they feel the need for a visual assessment, but this only works if patients are using a smartphone. Other solutions allow patients to use multiple devices at the same time. For example, they can use both a laptop and a smartphone to provide a clinician with multiple perspectives (e.g., they can aim a smartphone at their leg while monitoring the view on their laptop). Patients may even be asked to visit a local clinic or pharmacy where they can use special video consultation set-ups that have peripheral devices such as a stethoscope or oximeter, facilitating remote physical examinations. Interaction research urgently needs to pay attention to such technological infrastructure, what is available and to which patients, as well as how this shapes what they can and cannot do in a video consultation.

Second, interaction research needs to pay greater attention to how communication in video consultation is contextually situated (e.g., clinically, environmentally, socially, culturally). The rapid spread of video consulting has led to use across health care specialties (orthopedics, hematology, dermatology, primary care and so on), with the condition and clinical focus shaping wide-ranging use cases. These range from one-off primary care consultations between clinician and patient for a minor ailment (e.g., ear infection) through to repeated consultations between hospital-based clinicians, patients and carers regarding management of a long term condition (e.g., heart failure), and use of group video consultations with multiple patients and clinicians (e.g., to support smoking cessation). Use of video also varies by setting with differences, for instance, across primary and secondary care and in low- and middle-income countries compared to high income countries where technology, data and video-based services are (typically, but not always) more available. Finally, attention is needed on the immediate environment in which video consultations take place (e.g., the patients home or workplace), and allied resources that may shape the interaction (e.g., space, lighting, Wi-Fi). Each context (and combination of contexts) will have its own social and technological benefits and challenges that shape interaction, the quality of the service, and the experiences of the participants.

Third, research will also need to take account of the evolving nature of video consulting. The COVID-19 pandemic has led to rapid implementation of video consultations by healthcare providers, most of whom had little to no previous experience with this service model. As clinicians and patients gain experience, they will inevitably develop new routines and ways of working. These will be shaped by technological developments and wider infrastructure, as well as societal constraints, such as the push by national policymakers for a “digital first” or “remote-by-default” service model (NHS, 2019).^2^ Approaches like linguistic ethnography that can connect the evolution of technological, infrastructural and social developments with the back and forth of communication between clinicians and patients have a critical role to play in understanding this dynamic.

Finally, and relevant to all of the above, there is a need for mixed methods and interdisciplinary approaches. It is unlikely that individual studies alone can capture the richness needed to study the unfolding interaction of video consulting in situ as well as the social contexts shaping that interaction (and vice versa). This not only involves naturalistic observation of video consultations (ideally video-recorded at both “ends” of the consultation), but also direct observation of clinical routines and the settings in which consultations take place, as well as interviews with service users and policymakers that capture post-hoc reflections. It might also involve, for instance, access to patient records or other artefacts or technologies (e.g., remote monitoring systems) and the readouts and data they provide. It may well be that individual studies focus on one or two of these, with researchers then striving to integrate data and findings within and across studies. Interdisciplinary working is essential, enabling expertise, exchange and synthesis across, for instance, linguistics and language use, multimodality, ethnography, human-computer interaction and health services research.

Successful (and sustainable) spread and scale up of video consulting depends on adequate support for patients and clinicians who use the service (Greenhalgh et al., 2017). Methodological approaches like Linguistic Ethnography that use predominantly qualitative methods to study social context and interaction will be critical in helping researchers, funders and providers to make sense of how video consultations are accomplished, as well as how, when and why they fail. It will also inform development of guidance on communication in video consultations “that is effective for real-life practice and takes full account of the consultation as a co-constructed accomplishment” (Swinglehurst & Atkins, 2018, p. 410). Without this, it may be impossible to promote and maintain high quality healthcare services in an increasingly remote world.

## Notes

1. CA has its roots in Ethnomethodology, where researchers should strive to meet the “Unique Adequacy Requirement”: to be competent enough in the methods that members use, to be able to recognize and analyze these methods. Ethnographic methods, while not offering full-fledged training, allows us to acquire at least some of this competence.

2.
https://www.theguardian.com/society/2020/jul/30/all-gp-consultations-should-be-remote-by-default-says-matt-hancock-nhs
